# Bhopal: No Silver Linings

**DOI:** 10.1289/ehp.112-1247593

**Published:** 2004-10

**Authors:** J.P. Gupta, Kimberly Thigpen Tart

**Affiliations:** Department of Chemical Engineering, Indian Institute of Technology, Kanpur, India, E-mail: jpg@iitk.ac.in; News Editor, *EHP,* E-mail: thigpenk@niehs.nih.gov

I read with interest the article “Lessons Learned? Chemical Plant Safety since Bhopal,” by Ernie Hood (2004). I would recommend it to all interested in safety in chemical plants or safety in other fields.

As mentioned in the article (Hood 2004), this year is the 20th anniversary of the Bhopal tragedy. An international conference on the 20th anniversary of the tragedy, Bhopal and Its Effects on Process Safety, will be held 1–3 December 2004 at the Indian Institute of Technology in Kanpur, India, with a visit to the Bhopal plant planned on 4 December for those who are interested; details are available online (http://www.iitk.ac.in/infocell/announce/bhopal). Although the deadline for abstracts has passed, we will still consider outstanding papers.

I would also like to comment on the legend for the figure on page A354 of Hood’s article (Hood 2004): “A toxic cloud’s silver lining?” The question mark does indicate that there are some doubts whether the death of many thousands and the continued suffering of a still larger number should be considered to have a silver lining. In predictable accidents, the large number of deaths produce only untold suffering and not proportionate advantages to the society. The earlier leakages at the Union Carbide Bhopal plant were well known and documented in the newspapers, but neither the company nor the government took enough actions to save the city from the expected accident. According to Charles Perrow of Yale University (Perrow 1999), this is one accident that could not have been worse, contrasting the common cliche “we were lucky it wasn’t worse,” which is used to describe many other accidents and deliberate actions, such as the 9/11 attacks on the World Trade Center (WTC) in New York City. If the explosions in the WTC had taken place later in the day, many more people would have been inside the two towers and many more would have died. No one should say the deaths at the WTC and the Pentagon provide a silver lining to the war against terrorism. Terrorist acts were already being conducted in several places in Asia, Spain, Northern Ireland, Latin America, and other locations, except the world as a whole decided to look the other way and let individual countries respond. Similarly, because the problems caused by fascism were known or could be foreseen, World War II did not have to happen and cause many millions of deaths. The Allies recently observed the 60th anniversary of the D-Day invasions of several beaches in France; so many deaths and much misery was not necessary for us to understand what fascism could do.

Therefore, I hope that people would reconsider their comments of silver linings on others’ sufferings. The use of the question mark indicates that Hood (2004) was not sure of this, and I commend that hesitant punctuation mark.

## References

[b1-ehp0112-a0794a] Hood E (2004). Lessons learned? Chemical plant safety since Bhopal. Environ Health Perspect.

[b2-ehp0112-a0794a] Perrow C 1999. Normal Accidents. Princeton, NJ:Princeton University Press.

